# Therapeutic interventions and the length of hospital stay for pediatric patients with COVID-19: a multicenter cohort study

**DOI:** 10.1038/s41598-023-48904-w

**Published:** 2023-12-05

**Authors:** Tadashi Ishihara, Takashi Tagami, Atsushi Hirayama, Yuki Nakamura, Koichiro Sueyoshi, Ken Okamoto, Hiroshi Tanaka

**Affiliations:** 1 Department of Emergency and Critical Care Medicine, Juntendo University, Urayasu Hospital, 2-1-1, Tomioka, Urayasu-city, Chiba 279-0021 Japan; 2 Department of Emergency and Critical Care Medicine, Nippon Medical School Musashikosugi Hospital, Kawasaki-city, Kanagawa Japan; 3 Public Health, Department of Social Medicine, Graduate School of Medicine, Osaka University, Suita-city, Osaka Japan

**Keywords:** Epidemiology, Paediatric research

## Abstract

The evidence for pediatric patients with COVID-19 was very limited, which was attributed to the small number of the cases as well as the rare incidence of severe pneumonia in this population. This retrospective cohort study aimed to identify the characteristics of pediatric patients with COVID-19 in the early period of the pandemic by analyzing Diagnosis Procedure Combination (DPC) data in Japan. This retrospective cohort analysis of Japanese multicenter research on COVID-19 using DPC data compared the outcomes and costs of treatment for pediatric patients with COVID-19. Of 4700 patients with COVID-19, 186 pediatric patients were included in this study. Among the included pediatric patients, 17 received therapeutic drugs specifically for COVID-19, while the remaining 169 pediatric patients received only symptomatic therapy. There were no significant differences in the length of hospital stay (9 vs. 8 days, *p* = 0.96), and medical cost (97,585 vs. 73,291 JPY) for the intervention and control groups, respectively by multiple regression analysis. This is the first epidemiological study to use DPC data to summarize the pathophysiology of pediatric patients in the early period of COVID-19 pandemic. There was no significant difference in length of hospital stay or medical cost by intervention.

## Introduction

In December 2019, cases of pneumonia caused by a novel coronavirus, which was designated as severe acute respiratory coronavirus 2 (SARS-CoV-2), began to rapidly appear in Wuhan City in China. Subsequently, the pneumonitis associated with coronavirus disease 2019 (COVID-19) spread worldwide^1^. SARS-CoV-2 can spread easily among humans thorough droplets, and as it spread worldwide, the COVID-19 outbreak was certified as a pandemic by the World Health Organization^2^.

In the early period of the COVID-19 pandemic, the pneumonia was usually severe in elderly patients, and the mortality rate was high. On the other hand, the evidence for pediatric patients was very limited, which was attributable to the small number of pediatric cases with COVID-19 as well as the rare incidence of severe pneumonia in this population^3–6^. Although treatment regimens based on oral or intravenous antiviral medications and anti-inflammatory drugs such as steroids have been recently established for COVID-19, no established regimens were available in the early period of the pandemic, especially for pediatric patients; thus, pediatric patients received only symptomatic therapy^7,8^.

We hypothesized that “therapeutic interventions for pediatric patients with COVID-19 affect their clinical course.” This retrospective cohort study aimed to identify the characteristics of pediatric patients with COVID-19 in the early period of the pandemic by analyzing Diagnosis Procedure Combination (DPC) data and the medical records of participating institutes in Japan.

## Results

**Figure 1:**
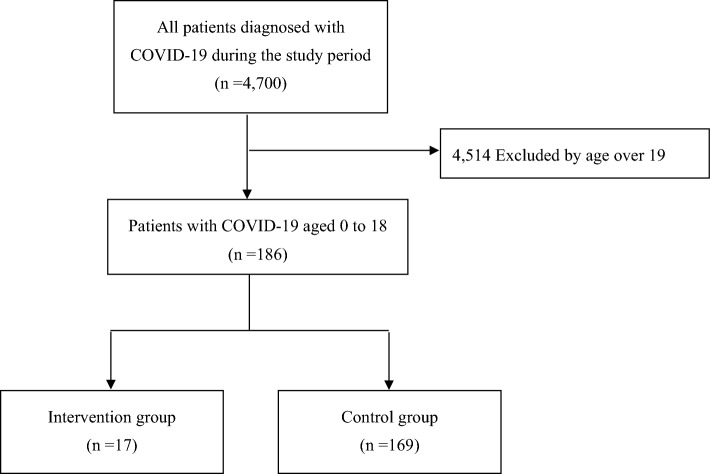
Inclusion criteria of the study. COVID-19: coronavirus disease 2019.

A total of 4700 COVID-19 patients with laboratory-confirmed SARS-CoV-2 infection from 66 participating hospitals were included in the main DPC study. Among the registered patients, 4,517 patients aged > 18 years were excluded from this study, and the remaining 186 pediatric patients (3.9%) were included in this sub-analysis of a Japanese multicenter COVID-19 study (Fig. 1). Among the included pediatric patients, 17 received therapeutic drugs specifically for COVID-19, while the remaining 169 pediatric patients received only symptomatic therapy and were categorized as the control group.

Patient demographics and characteristics are presented in Table 1. The median age of the intervention and control groups was 1 and 9 years, respectively; the intervention group was significantly younger than the control group (*p* < 0.05). The two groups also showed significant differences in age, transportation from another hospital (*p* < 0.05), transportation by ambulance (*p* < 0.05), symptoms of dyspnea (*p* < 0.05), disturbance of consciousness (*p* < 0.05), and administration of oxygen (*p* < 0.05).

**Table 1:** Patient’s characteristics.

Characteristics	Intervention group (n = 17)	Control group (n = 169)	*p*-values
Age (years)	1 (0–5)	9 (2–14)	< 0.05*
Gender (male, %)	12 (70.6)	88 (52.1)	0.20
Urgent admission (%)	17 (100)	160 (95.8)	1
Transfer from another hospital	7 (41.2)	30 (17.8)	< 0.05*
Ambulance (%)	5 (29.4)	14 (8.3)	< 0.05*
Rase (%)			0.16
Japanese	16 (94.1)	139 (82.2)	
Asia (non-Japanese)	0	9 (5.3)	
White	1 (5.9)	0	
Black	0	2 (1.2)	
Latin	0	16 (9.5)	
Others	0	3 (1.8)	
Dyspnea (%)	4 (23.5)	3 (1.8)	< 0.05*
Vital signs (median, IQR)			
BP (mmHg)	119 (106–131)	110 (98–118)	0.08
HR (beats/min)	110 (79–139)	91 (78–110)	0.15
RR (/min)	22 (18–30)	18 (16–24)	0.12
BT (℃)	37.5 (36.37.5)	36.8 (36.5–37.2)	0.06
SpO_2_ (%)	98 (97–99)	98 (97–99)	0.79
GCS < 15 (%)	2 (11.8)	0	< 0.05*
Oxygenation	2 (11.8)	1 (0.6)	< 0.05*

* *p* < 0.05.

IQR: Interquartile range, BP: Blood pressure, HR: Heart rate, RR: Respiratory rate.

BT: Body temperature, GCS: Glasgow coma scale.

Table 2 shows a list of treatments in the intervention group. The most commonly administered drug to pediatric patients was heparin (9,52.1%), followed by antimicrobial agents (8,47.1%). Corticosteroids, which are strongly recommended therapy for COVID-19, were only administered to two patients (1.1% of all pediatric patients). Remdesivir, as an antiviral agent, was administered to only one patient, i.e., only 0.5% of all pediatric patients.

**Table 2:** Intervention.

Medication	n = 17
Heparin	9 (52.9%)
Antimicrobial agent	8 (47.1%)
Favipiravir	2 (11.8%)
Remdesivir	1 (5.9%)
Corticosteroid	2 (11.8%)

The blood test results obtained on admission are presented in Table 3. The median white blood cell counts were 8900/µL and 5100/µL in the intervention and control groups, respectively (*p* < 0.05). C-reactive protein (CRP) levels were significantly higher in the intervention group than in the control group (0.37 vs. 0.07, *p* < 0.05). Blood albumin and sodium levels were significantly lower in the intervention group than in the control group. No bacteria was detected from the culture in the both group.

**Table 3:** Laboratory data on admission.

Laboratory data	Intervention group (n = 17)	Control group (n = 169)	*p*-values
WBC (× 10^3^/ul)	8.9 (5.1–11.5)	5.1 (4.0–7.8)	< 0.05*
Lympho (%)	46.5 (33.8–56.8)	35 (26.5–44.3)	0.18
Hct (%)	37.1 (34.0–42.5)	41.2(38.1–43.7)	0.05
Plt (× 10^4^/ul)	25.5 (19.5–32.9)	22.9 (20.3–27.0)	0.55
Alb (g/dl)	4.1 (3.9–4.5)	4.5 (4.3–4.8)	< 0.05*
AST (U/l)	39 (28–40)	20 (17–32)	< 0.05*
ALT (U/l)	18 (14–29)	13 (10–18.5)	0.09
LDH (U/l)	267 (192–296)	197 (169–256)	0.18
CK (mg/dl)	104 (82–109)	84 (55–149)	0.36
BUN (mg/dl)	9.0 (6.6–13.2)	11.6 (9.5–13.7)	0.17
Cre (mg/dl)	0.25 (0.22–0.76)	0.56 (0.39–0.75)	0.10
Na (mEq/l)	139 (138–140)	141 (139–141)	< 0.05*
K (mEq/l)	4.3 (4.0–4.5)	4.2 (4.0–4.4)	0.33
Glu (mg/dl)	93 (83–98)	96 (86–105)	0.50
Lac (mmol/l)	1.8 (1.7–2.7)	2.0 (1.8–2.3)	1
CRP (mg/dl)	0.37 (0.13–1.07)	0.07(0.02–0.22)	< 0.05*
Procalcitonin (ng/ml)	0.35 (0.08–22.07)	0.04 (0.02–0.09)	0.05
Ferritin (ng/ml)	197 (88–333)	49 (35–109)	0.21
PT-INR	1.09 (0.99–1.24)	1.00 (0.97–1.09)	0.26
APTT (sec)	31.5 (72.8–39.1)	32.4 (29.4–36.3)	0.74
D-dimer (μg/ml)	0.5 (0–0.7)	0.5 (0.3–0.8)	0.58
FDP (μg/ml)	0 (0–1.15)	0 (0–2.5)	0.66
Fibrinogen (mg/dl)	251 (241–320)	283 (254–300)	0.66
AT III (%)	82 (74–89)	114 (112–124)	0.06
HbA1c (%)	5.3 (5.2–5.3)	5.5 (5.2–5.6)	0.33

* *p* < 0.05.

WBC: white blood cell, AST: asparate aminotransferase, ALT: alanine aminotransferase, LDH: lactate dehydrogenase, CK: creatinine kinase, BUN: blood urea nitrogen,

CRP: C-reactive protein, PT-INR: prothrombin time-international normalized ratio,

APTT: activated partial thromboplastin time, FDP: fibrin degradation product,

AT III: antithrombin III, HbA1c: hemoglobin A1c.

Table 4 lists the patient outcomes. Three patients died during the study period, and the mortality rates did not differ significantly between the groups. The median length of hospital stay was 9 and 8 days for the intervention and control groups, respectively (*p* = 0.92). The two groups showed no significant differences in the length of hospital stay. The two groups showed a significant difference only in medical cost (97,585 vs. 75,382; *p* < 0.05). None of the patients showed multisystem inflammatory syndrome in children (MIS-C) complications.

**Table 4:** Patient’s outcome.

Outcomes	Intervention group (n = 17)	Control group (n = 169)	*p*-values
Length of hospital stay (days), median (IQR)	9 (5–10)	8 (7–11)	0.92
Medical cost (JPY)	97,585	75,382	< 0.05*
median (IQR)	(64,754–130,758)	(45,804–113,555)	
Mortality (%)	1 (5.9)	2 (1.2)	0.25
Discharge (%)	16 (94.1)	167 (98.8)	0.25
Complication (%)			
Hemorrhage	0	1 (0.6)	1
MIS-C	0	0	1

* *p* < 0.05.

IQR: Interquartile range, JPY: Japanese yen, MIS-C: Multisystem inflammatory syndrome in children.

The results of the multiple regression analysis for the length of hospital stay and medical cost are shown in Table 5. There was no significant difference in length of hospital stay and medical cost between the two groups, matched by age and severity of COVID-19.

**Table 5:** Multiple regression analysis.

Outcomes	Intervention group (n = 17)	Control group (n = 169)	*p*-values
Length of hospital stay (days), median (IQR)	9 (5–10)	8 (7–10)	0.96
Medical cost (JPY)	97,585	73,291	0.17
median (IQR)	(64,754–130,758)	(49,723–106,76-)	

* *p* < 0.05.

IQR: Interquartile range, JPY: Japanese yen.

## Discussion

This was an epidemiological study of pediatric patients with COVID-19 that analyzed DPC data during the early period of the COVID-19 pandemic. Although this was a multicenter retrospective study including adult patients, the number of pediatric patients aged < 19 years was very small, accounting for only 3.8% of all patients. Reports from the Centers for Disease Control and Prevention (CDC) have shown that pediatric patients accounted for only 1.7% of the total cases in the USA, 4% of those in Australia, and 1.2% of those in Italy during similar period^9–11^. Pediatric patients accounted for only 2% of the cases in China, the origin of the COVID-19 pandemic^12^, which is not substantially different from the corresponding value in our report.

In contrast to adult cases, most pediatric patients with COVID-19 show mild clinical symptoms, and some pediatric patients show no obvious clinical manifestations ^4–6,13–16^. In this study, only three patients required oxygen administration, accounting for 1.6% of all pediatric patients with COVID-19. Moreover, only 1.7% of pediatric patients showed severe disease^13,17–19^. Death among pediatric patients, one of the outcomes of the study, was rare, with only three patients dying during the study period. This study included only in-hospital pediatric patients; if outpatients were included, the mortality rate would be extremely low. Reports from other countries also show that mortality rates for pediatric patients are quite low^20^. On the other hand, approximately 3% to 15% of adult patients with COVID-19 show deterioration to severe disease^20^.

The laboratory test and DPC data of pediatric patients with COVID-19 in this study indicate that the condition of pediatric patients with COVID-19 generally seems to be relatively mild, similar to the findings of other reports. The presumed reasons for the generally mild presentation in pediatric cases can be summarized as follow: the angiotensin-converting enzyme-2 (ACE2) receptor of children’s lungs is still immature than that of adults. SARS-CoV-2 invades the epithelial cells of the airway and proliferates through the ACE-2 receptor on the cell surface. Thus, the immaturity of the ACE-2 receptor in children enhances their resistance to COVID-19^21,22^.

Most pediatric patients with COVID-19 show a mild disease course and improve with symptomatic therapy only, and therapeutic interventions are rarely required^6^. Although a few reports have evaluated pediatric cases with COVID-19, only a few case reports from the early periods of the pandemic have been published to date. Reports from China or Malaysia indicate that 17–40% of pediatric patients with COVID-19 received lopinavir/ritonavir^23–25^. One report from Malaysia indicated that approximately 50% of pediatric patients received paracetamol, 25% received antibiotics, and 25% received salbutamol^26^. One report of 200 pediatric COVID-19 patients from a tertiary children’s hospital in Portugal indicated that only 37 patients were admitted due to COVID-19, and four of them were admitted to the intensive care unit (ICU). Twenty patients (59%) received antiviral drugs, whereas 27% only received symptomatic therapy. Almost all patients showed improvement within 24–72 h. Eight patients received lopinavir/ritonavir, 3 received remdesivir, and 16 received antibiotics. Among the in-hospital patients, seven received oxygen therapy, and three (1.5%) received mechanical ventilation. Only one patient was diagnosed with MIS-C^27^. The study periods in these reports were similar to that of our study, and the results of our study are similar to those of other reports.

As the pandemic lengthened, treatment options for COVID-19 were gradually established, including steroids, remdesivir, or heparin, but no established therapy was available for pediatric patients with COVID-19 during the study period^28^. No definitive treatment was available for pediatric patients with COVID-19, and they had to be treated with antipyretics for symptomatic therapy. In our study, only 9.1% of the patients received heparin, antiviral drugs such as remdesivir or lopinavir/ritonavir, or antibiotics. Additionally, patients in the intervention group were significantly younger than those in the control group. Infants with sepsis or infectious disease are considered to have a high risk of deterioration, and thus required aggressive therapeutic intervention^29^. However, the indication for the therapeutic intervention could not be clearly, because the DPC data did not include that information. The two groups showed no significant differences in the length of hospital stay or medical cost by multiple regression analysis.

In contrast to adult patients, inflammatory markers often show a normal range in pediatric patients with COVID-19^30^. In our study, inflammatory markers, that is, CRP, procalcitonin, or ferritin, were tested at admission and were almost within the normal range. The levels of inflammatory markers were not elevated in pediatric patients with COVID-19, probably because pediatric cases are less severe and resolve with mild disease.

Our study had several limitations. First, we conducted a retrospective analysis; therefore, only associations among available data could be described. Second, the DPC data did not include information relevant to the definitive indications for therapeutic interventions. Moreover, the indications for therapeutic interventions depend on each participating institute along with the patient’s condition or severity, and DPC data did not provide institutional characteristics, potentially resulting in an institutional bias. Third, the number of pediatric patients with COVID-19 were extremely smaller than adult patients, so there is concern in the statistical power. Additionally that, due to the rapid evolution of viral strains, it should be noted that the generalizability of this study may be limited. Fourth, there were cases of missing data, and this might have generated information bias. Finally, the DPC data did not cover all pediatric patients in Japan, and this study may have a selection bias if a disproportionately greater number of academically focused institutes participated in it.

In conclusion, this epidemiological study analyzing DPC data of pediatric patients with COVID-19 during the early period of the COVID-19 pandemic showed no significant difference in the length of hospital stay, medical cost or mortality between patients who received interventions for COVID-19 and those who received symptomatic treatment. Only a few patients received therapy relevant to COVID-19, and most patients recovered from COVID-19 without specific therapy.

This study is the first epidemiological study to use DPC data to summarize the pathophysiology of pediatric patients in the early period of COVID-19 pandemic, and is important for understanding the pathogenesis of COVID-19.

## Methods

### Study setting and participants

This study was a sub-analysis of Japanese multicenter research on COVID-19 performed by assembling real-world data (J-RECOVER study)^31^. The study adhered to the protocol described in a previous report^31^. The original survey involved collection of patient information from DPC data and medical records at each participating institute. Pediatric patients aged < 19 years with COVID-19 who had laboratory-confirmed SARS-CoV-2 infection by PCR and were discharged from the participating hospital between January 1, 2020, and September 31, 2020, were included in this study.

This retrospective cohort analysis of Japanese multicenter research on COVID-19 using DPC data compared the outcomes and costs of treatment for pediatric patients with COVID-19. Patients were divided into two group. The intervention group was defined as the group that received antiviral agents, antimicrobial agents, or other medication, specific for COVID-19, and the control was the group that received only symptomatic treatment.

### Ethical information

This study was approved by the Institutional Review Board of Juntendo University Urayasu Hospital, Chiba, Japan (Ethic Committee of Juntendo University/ U20-0026) and was conducted in accordance with the principles outlined in 1964 Declaration of Helsinki and its later amendments. The need for informed consent was waived by the Institutional Review Board of Juntendo University, Urayasu Hospital, Chiba, Japan owing to the retrospective nature of the study. We followed the STROBE reporting guidelines while conducting this study.

In this study, the patients’ clinical information was obtained from the DPC data and medical records at each participating institute. Other information relevant to the research problem that could not be obtained from the DPC data was obtained as “additional clinical information” from the medical records. Facility and laboratory data were also collected on admission and provided by each hospital. Blood data and urine results obtained during hospitalization or on admission, and initial culture results were collected and provided by each hospital.

### DPC data

A diagnosis group classification system based on the DPC was introduced in acute care hospitals in 2003, and administrative claim data were created and stored electronically at each facility under a comprehensive payment system based on the DPC^32^. The DPC system started with 82 academic hospitals, and subsequently grew to include more than 1600 participating acute care hospitals that are submitting DPC data to the Ministry of Health, Labour and Welfare in Japan^32^. The DPC data includes information regarding the patient’s sex, birth date, main diagnosis of admission, admission and discharge date, admission route, scheduled or urgent admission, discharge destination, and discharge outcome. The main diagnosis, disease that led to admission, and comorbidities at hospitalization are all coded according to the International Classification of Disease, 10th Revision. In addition to the Japan Coma Scale scores at admission, all medical and surgical procedures and records of all prescribed drugs and devices are also included in the DPC data^33,34^. The DPC data file for each month at each hospital is generated in a single file, which contains data for all patients from all departments discharged in that month, and is submitted to the Ministry of Health, Labour and Welfare.

### Outcome measures

The primary outcome was the length of hospital stay. The secondary outcomes were complications such as MIS-C, hemorrhage, medical costs, and mortality.

### Statistical analysis

Patient data displayed as the median with interquartile range (IQR) were used as numeric variables. Baseline patient characteristics were compared using a chi-square test or Fisher’s exact test for frequencies and* t*-test or Mann–Whitney *U*-test for continuous variables, as appropriate. Differences were considered significant when the *P-*value was less than 0.05. ^35^ To assess the independent effect of the study, multivariable regression analysis was performed on the length of hospital stay and medical cost. Age and severity of COVID-19 were included as variables in the analysis. Data management and statistical analysis were performed using the EZR software (Y Kaneda, Saitama Medical Centre, Jichi Medical University, Saitama Japan).

## Data Availability

The data sets generated and/or analyzed during the current study are publicly available due to including of privacy but are available from the corresponding author on reasonable request.

## Abbreviations

SARS-CoV-2: Severe acute respiratory coronavirus 2
COVID-19: Coronavirus disease 2019
DPC: Diagnosis procedure combination
CRP: C-reactive protein
MIS-C: Multisystem inflammatory syndrome in children
CDC: Centers for disease control and prevention
ACE-2: Angiotensin-converting enzyme-2
ICU: Intensive care unit
PCR: Polymerase chain reaction
IQR: Interquartile range

## References

[1] Zhu N (2020). A novel coronavirus from patients with pneumonia in China, 2019. N. Engl. J. Med..

[2] Cucinotta D, Vanelli M (2020). WHO declares COVID-19 a pandemic. Acta Biomed..

[3] Alsohime F, Temsah MH, Al-Nemri AM, Somily AM, Al-Subaie S (2020). COVID-19 infection prevalence in pediatric population: Etiology, clinical presentation, and outcome. J. Infect. Public Health.

[4] Qi K (2021). Clinical, laboratory, and imaging features of pediatric COVID-19: A systematic review and meta-analysis. Medicine (Baltimore).

[5] Mehta NS (2020). SARS-CoV-2 (COVID-19): What do we know about children? A systematic review. Clin. Infect. Dis..

[6] Hoang A (2020). COVID-19 in 7780 pediatric patients: A systematic review. EClinicalMedicine.

[7] Panda PK (2021). COVID-19 treatment in children: A systematic review and meta-analysis. J. Fam. Med. Prim. Care.

[8] Irfan O (2021). Clinical characteristics, treatment and outcomes of paediatric COVID-19: A systematic review and meta-analysis. Arch. Dis. Child..

[9] Disease C (2019). in Children–United States, February 12-April 2, 2020. MMWR Morb. Mortal Wkly. Rep..

[10] COVID-19, Australia: Epidemiology Report 11 (Reporting week to 23:59 AEST 12 April 2020). Commun Dis Intell (2018) **44**, 10.33321/cdi.2020.44.34 (2020). 10.33321/cdi.2020.44.34 32299330

[11] Livingston E, Bucher K (2020). Coronavirus disease 2019 (COVID-19) in Italy. JAMA.

[12] Wu Z, McGoogan JM (2020). Characteristics of and important lessons from the coronavirus disease 2019 (COVID-19) outbreak in China: Summary of a report of 72 314 cases from the Chinese center for disease control and prevention. JAMA.

[13] Lu X (2020). SARS-CoV-2 infection in children. N. Engl. J. Med..

[14] Rodriguez-Morales AJ (2020). Clinical, laboratory and imaging features of COVID-19: A systematic review and meta-analysis. Travel Med. Infect. Dis..

[15] Huang C (2020). Clinical features of patients infected with 2019 novel coronavirus in Wuhan, China. Lancet.

[16] Gudbjartsson DF (2020). Spread of SARS-CoV-2 in the Icelandic population. N. Engl. J. Med..

[17] Götzinger F (2020). COVID-19 in children and adolescents in Europe: A multinational, multicentre cohort study. Lancet Child Adolesc. Health.

[18] Kollmann TR (2009). Neonatal innate TLR-mediated responses are distinct from those of adults. J. Immunol..

[19] Kollmann TR, Kampmann B, Mazmanian SK, Marchant A, Levy O (2017). Protecting the newborn and young infant from infectious diseases: Lessons from immune ontogeny. Immunity.

[20] Xu T (2020). Clinical features and dynamics of viral load in imported and non-imported patients with COVID-19. Int. J. Infect. Dis..

[21] Cao Y (2020). Comparative genetic analysis of the novel coronavirus (2019-nCoV/SARS-CoV-2) receptor ACE2 in different populations. Cell Discov..

[22] Lu R (2020). Genomic characterisation and epidemiology of 2019 novel coronavirus: Implications for virus origins and receptor binding. Lancet.

[23] Zhu L (2020). Clinical characteristics of a case series of children with coronavirus disease 2019. Pediatr. Pulmonol..

[24] Qiu H (2020). Clinical and epidemiological features of 36 children with coronavirus disease 2019 (COVID-19) in Zhejiang, China: An observational cohort study. Lancet Infect. Dis..

[25] Song W (2020). Clinical features of pediatric patients with coronavirus disease (COVID-19). J. Clin. Virol..

[26] See KC (2020). COVID-19: Four paediatric cases in Malaysia. Int. J. Infect. Dis..

[27] Saraiva BM, Garcia AM, Silva TM, Gouveia C, Brito MJ (2021). Clinical and therapeutic approach to hospitalized COVID-19 patients: A pediatric cohort in Portugal. Acta Med. Port..

[28] Yamakawa K (2021). Japanese rapid/living recommendations on drug management for COVID-19: Updated guidelines (September 2021). Acute Med. Surg..

[29] Weiss SL (2020). Surviving sepsis campaign international guidelines for the management of septic shock and sepsis-associated organ dysfunction in children. Intensive Care Med..

[30] Henry BM, Lippi G, Plebani M (2020). Laboratory abnormalities in children with novel coronavirus disease 2019. Clin. Chem. Lab. Med..

[31] Tagami T, Yamakawa K, Endo A (2021). Japanese multicenter research of COVID-19 by assembling real-world data: A study protocol. Ann. Clin. Epidemiol..

[32] Yasunaga H (2019). Real world data in Japan: Chapter II the diagnosis procedure combination database. Ann. Clin. Epidemiol..

[33] Tagami T, Matsui H, Fushimi K, Yasunaga H (2015). Low-dose corticosteroid treatment and mortality in refractory abdominal septic shock after emergency laparotomy. Ann. Intensive Care.

[34] Tagami T (2017). Antithrombin use and 28-day in-hospital mortality among severe-burn patients: An observational nationwide study. Ann. Intensive Care.

[35] Segerstrom SC (2019). Statistical guideline #2: Report appropriate reliability for your sample, measure, and design. Int. J. Behav. Med..

