# Author Correction: Therapeutic interventions and the length of hospital stay for pediatric patients with COVID-19: a multicenter cohort study

**DOI:** 10.1038/s41598-024-62167-z

**Published:** 2024-06-11

**Authors:** Tadashi Ishihara, Takashi Tagami, Atsushi Hirayama, Yuki Nakamura, Koichiro Sueyoshi, Ken Okamoto, Hiroshi Tanaka

**Affiliations:** 1 Department of Emergency and Critical Care Medicine, Juntendo University, Urayasu Hospital, 2-1-1, Tomioka, Urayasu-City, Chiba 279-0021 Japan; 2 Department of Emergency and Critical Care Medicine, Nippon Medical School Musashikosugi Hospital, Kawasaki-City, Kanagawa Japan; 3 Public Health, Department of Social Medicine, Graduate School of Medicine, Osaka University, Suita-City, Osaka Japan

Correction to: *Scientific Reports* 10.1038/s41598-023-48904-w, published online 05 December 2023

Atsushi Hirayama and Takashi Tagami were omitted from the author list in the original version of this Article.

The Author Contributions section now reads:

“All authors contributed to the study conception and design. T.T. and A.Y. compiled the J-RECOVER study data and provided advice on the study. Data collection and analysis were performed by T.I., Y.N. and K.S. The first draft of the manuscript was written by T.I. H.T. and K.O. provided critical review. All authors read and approved final manuscript.”

In addition, in the Results section,

“A total of 4700 COVID-19 patients with laboratory-confirmed SARS-CoV-2 infection from 62 participating hospitals were included in the main DPC study.”

now reads:

“A total of 4700 COVID-19 patients with laboratory-confirmed SARS-CoV-2 infection from 66 participating hospitals were included in the main DPC study.”

“The most commonly administered drug to pediatric patients was heparin (952.1%), followed by antimicrobial agents (847.1%).”

now reads:

“The most commonly administered drug to pediatric patients was heparin (9,52.1%), followed by antimicrobial agents (8,47.1%).”

In the Methods section, under the subheading ‘Study setting and participants’,

“This study was a sub-analysis of Japanese multicenter research on COVID-19 performed by assembling real-world data^31^.”

now reads:

“This study was a sub-analysis of Japanese multicenter research on COVID-19 performed by assembling real-world data (J-RECOVER study)^31^”.

Under the subheading, ‘DPC data’,

“A diagnosis group classification system based on the DPC was introduced in acute care hospitals in 2022, […]”

now reads:

“A diagnosis group classification system based on the DPC was introduced in acute care hospitals in 2003, […]”.

The original Article has been corrected.

